# Screening candidate microRNA-mRNA regulatory pairs for predicting the response to chemoradiotherapy in rectal cancer by a bioinformatics approach

**DOI:** 10.1038/s41598-017-11840-7

**Published:** 2017-09-12

**Authors:** Qiliang Peng, Junjia Zhu, Peipei Shen, Wenyan Yao, Yu Lei, Li Zou, Yingying Xu, Yuntian Shen, Yaqun Zhu

**Affiliations:** 1 Department of Radiotherapy & Oncology, The Second Affiliated Hospital of Soochow University, Suzhou, China; 2 Institute of Radiotherapy & Oncology, Soochow University, Suzhou, China; 3 Suzhou Key Laboratory for Radiation Oncology, Suzhou, China; 4 Suzhou Medical Center of Radiotherapy & Oncology, Suzhou, China; 5 Department of Anorectal Surgery, The Affiliated Jiangyin Hospital of Southeast University Medical College, Jiangyin, China; 6 Department of General Surgery, The Second Affiliated Hospital of Soochow University, Suzhou, China

## Abstract

Extensive efforts have been undertaken in search of biomarkers for predicting the chemoradiotherapy response in rectal cancer. However, most attention on treatment efficiency prediction in carcinoma is addicted to single or limited molecules. Network biomarkers are considered to outperform single molecules in predictive power. In this study, candidate microRNAs (miRNAs) were identified from the PubMed citations and miRNA expression profiles. Targets of miRNAs were obtained from four experimentally confirmed interactions and three computationally predicted databases. Functional enrichment analysis of all the targets revealed their associations with chemoradiotherapy response, indicating they could be promising biomarkers. Two lists of key target mRNAs of the candidate miRNAs were retrieved from protein–protein interaction (PPI) network and mRNA expression profiles, respectively. Pathway analysis and literature validation revealed that the mRNA lists were highly related to the ionizing radiation. The above miRNAs along with the key miRNA targets provide potential miRNA-mRNA regulatory pairs as network biomarkers in which all the network components may be used for predicting the chemoradiotherapy response. These results demonstrated that the network biomarkers could provide a useful model for predicting the chemoradiotherapy response and help in further understanding the molecular basis of response differences, which should be prioritized for further study.

## Introduction

Rectal cancer remains one of the most commonly diagnosed malignancies with leading mortality worldwide^1^. Preoperative chemoradiotherapy is currently considered to be a standard treatment procedure for improving local control of the disease, preserving sphincter and thus has a benefit in regards to survival^2^. Nevertheless, tumor responses to the treatment modality differ among individuals^3^. Therefore, exploring biomarkers for early response prediction before treatment would help in proceeding with avoiding potentially nonresponsive patients from unnecessary treatment with possible accompanying side effects.

During the past decades, an increasing number of studies have been previously investigated the biomarkers in attempts to predict the response to chemoradiotherapy. Up to now, several genetic and molecular biomarkers have been identified with the prediction potential including epidermal growth factor receptor^4^, thymidylate synthase^5^, Bax^6^, Bcl-2^7^, p53^8^ and p21^9^. Despite the progress achieved, there still has been no specific molecular marker proven to predict the response to chemoradiotherapy and uncover the potential mechanisms for the difference in radiosensitivity among patients due to the controversial and inconclusive results. In recent years, microRNAs (miRNAs), small noncoding RNAs, playing vital roles in regulating mRNA expression, have been reported to hold the ability to distinguish responders from non-responders to preoperative chemoradiotherapy^10, 11^. Furthermore, it is gratifying that recent technology progresses in expression genomics by DNA microarray have made it possible to complete the simultaneous analysis of a great deal of miRNAs and mRNAs and may be used for the systematic search for molecular markers^12^. Accordingly, dealing with a very wide range of complexly structured data types of miRNA and mRNA and combine them into a strong theoretical framework may further help for the biomarkers discovery.

In contrast to traditional biomarker researches with an isolated and static mode that focus on individual molecules, in this study, we integrated the miRNA expression information, mRNA expression data, miRNA-mRNA regulatory network, protein-protein interaction data, and other types of genomic information into a systems biological analysis in order to explore the network biomarkers in terms of miRNA-mRNA regulatory pairs for predicting the rectal cancer responses to chemoradiotherapy. We would like to uncover the potential prediction mechanism underlying these biomarkers with the use of several bioinformatics approaches. The findings from our analysis may have implications for predicting the chemoradiotherapy outcome and enable us to light the further insight for the mechanisms underlying the radiosensitivity difference.

## Results

### Detection of candidate biomarker miRNAs

**Figure 1:**
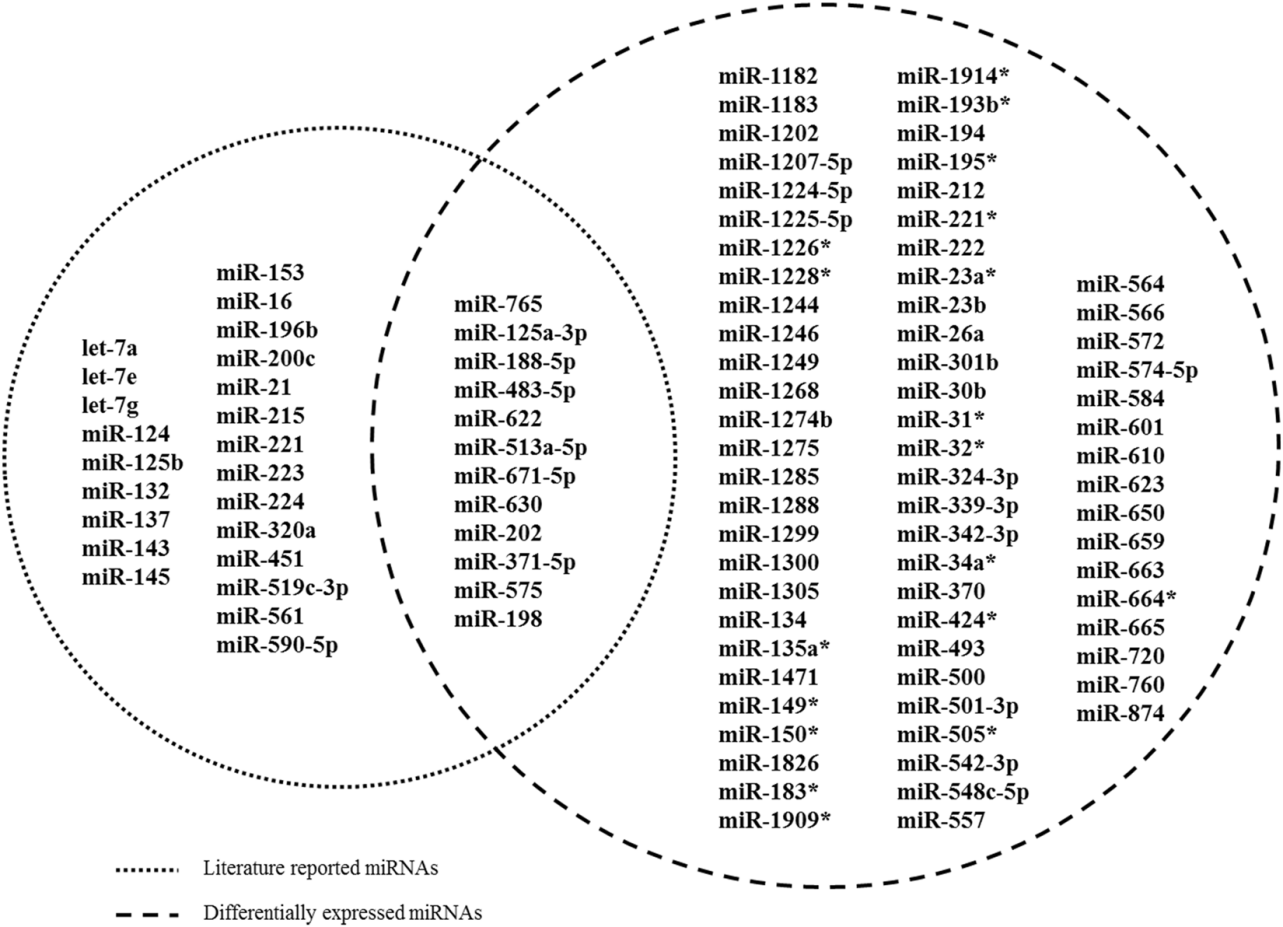
Venn diagram for literature-reported chemoradiotherapy related miRNAs and DE miRNAs. Dashed circles on the left and right represent literature-reported miRNAs and DE miRNAs, respectively.

By a thorough search in PubMed, we collected 35 miRNAs that may be helpful for distinguishing responders from non-responders to preoperative chemoradiotherapy. Furthermore, we exploited miRNA expression profiles to investigate the expression information. As described in Methods, we screened 82 significantly miRNAs differentially expressed (DE) between the responders and non-responders to preoperative chemoradiotherapy from the chosen miRNA expression dataset (GSE29298). Finally, after taking the intersection, we identified 12 candidate miRNA biomarkers (Fig. 1). The details of the 12 candidate miRNA biomarkers were listed at Table 1.

**Table 1:** Details of candidate miRNA biomarkers.

Reported ID	Official ID	P-value (responders versus non-responders)	Number of targets
miR-765	hsa-miR-765	1.39E-05	98
miR-125a-3p	hsa-miR-125a-3p	5.44E-05	36
miR-188-5p	hsa-miR-188-5p	9.38E-05	16
miR-483-5p	hsa-miR-483-5p	1.44E-04	74
miR-622	hsa-miR-622	1.91E-04	3
miR-513a-5p	hsa-miR-513a-5p	3.01E-04	23
miR-671-5p	hsa-miR-671-5p	5.96E-04	68
miR-630	hsa-miR-630	9.69E-04	121
miR-202	hsa-miR-202	1.40E-03	82
miR-371-5p	hsa-miR-371-5p	2.11E-03	63
miR-575	hsa-miR-575	4.07E-03	65
miR-198	hsa-miR-198	6.80E-03	174

### Targets prediction and functional enrichment

The target mRNAs of miRNAs were obtained by integrating experimentally proved and computationally predicted miRNA–mRNA interactions. It comprised 823 links between 12 miRNAs and 758 mRNAs. Detailed lists of miRNAs and their regulatory mRNAs are available in Supplementary Table S1.

**Figure 2:**
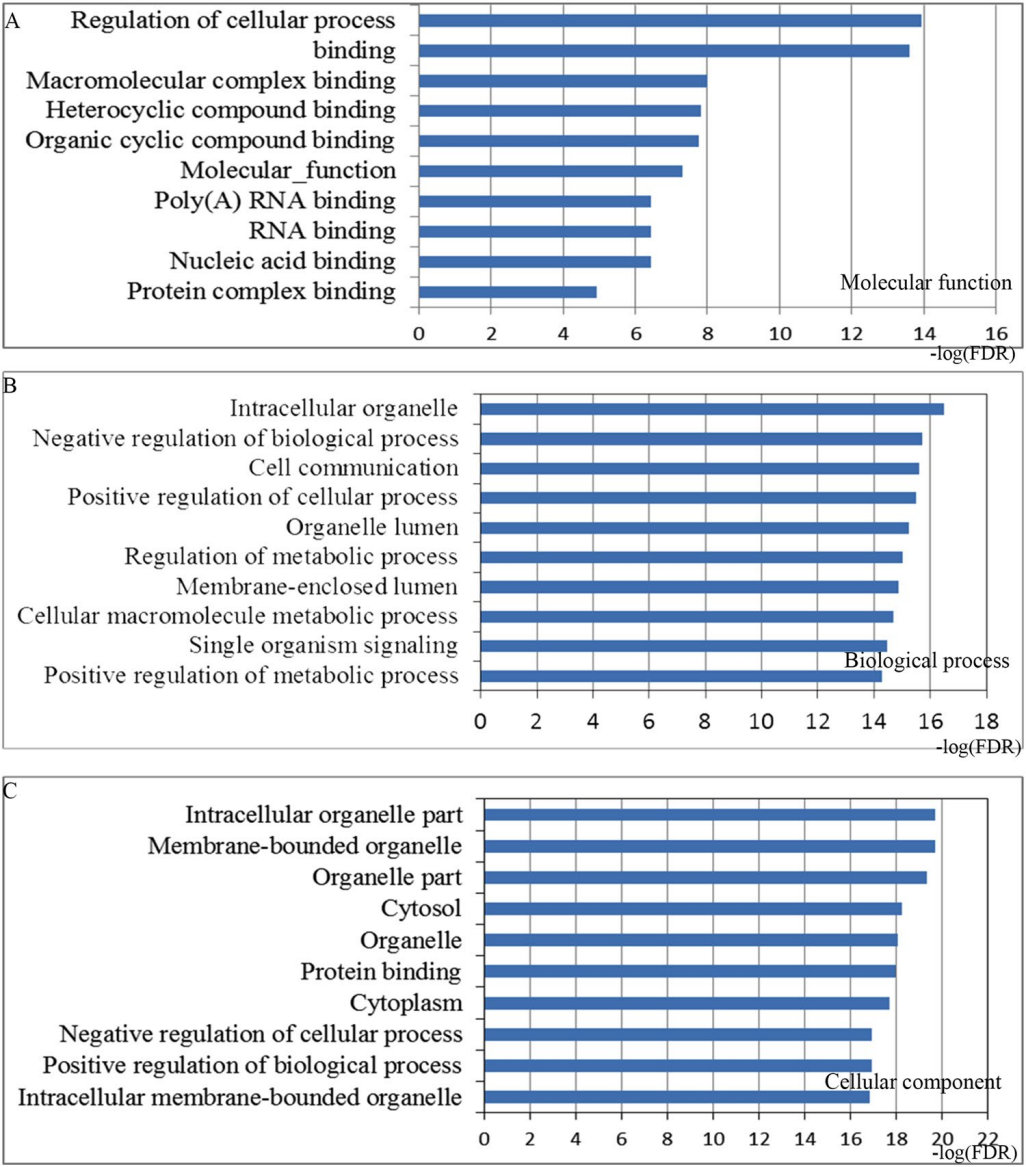
Gene ontology (GO) annotations for the targets of identified miRNA biomarkers. The targeted mRNAs by identified biomarker miRNAs were annotated by STRING at three levels, including molecular function (**A**), biological process (**B**) and cellular component (**C**). The top 10 significantly enriched items for each domain are shown.

**Figure 3:**
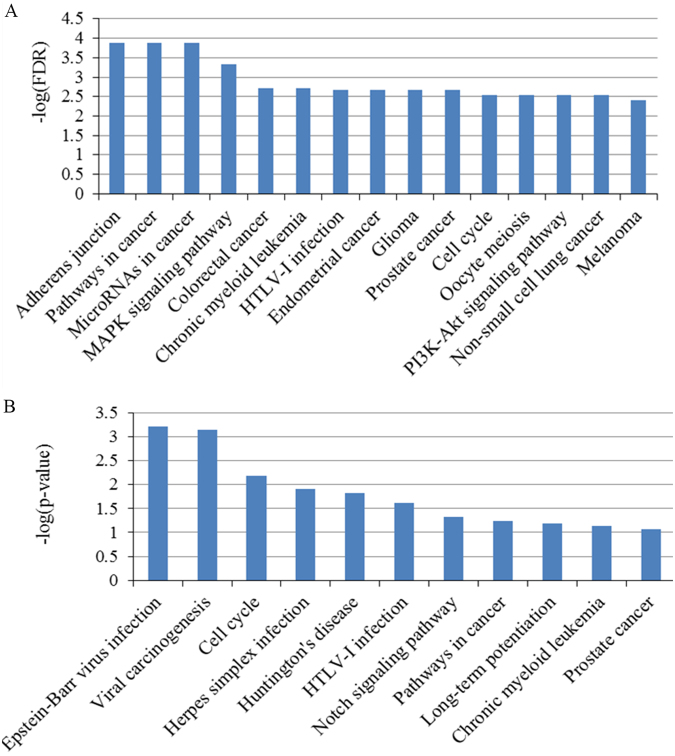
KEGG pathway enrichment analysis results. (**A**) Top 15 pathways enriched by all the targets of identified miRNA biomarkers. (**B**) Pathways enriched by the ten hub mRNAs from the PPI network.

To make a thorough inquiry for the function of the candidate miRNA markers and investigate their involved signaling pathways in chemoradiotherapy response, we accomplished the functional enrichment by mapping all their target mRNAs to the Search Tool for the Retrieval of Interacting Genes (STRING) database. The gene ontology (GO) enrichment results were illustrated the function of these target mRNAs at three different levels including molecular function (MF), cell component (CC) and biological processes (BP). The top 10 items significantly enriched by the target mRNAs for each of the three GO levels were shown in Fig. 2. Pathways further revealed their mechanisms and the top 15 significantly enriched terms are plotted in Fig. 3. Detailed lists of all the significantly enriched pathways and the matching mRNAs of the top 15 enriched categories could be found in Supplementary Tables S2 and S3.

### Identification of chemoradiotherapy response related functions and pathways

Based on the above results, we carefully screened the enriched functional categories and pathways by manually mining citations in PubMed.

The enriched GO terms in BP mainly included the regulation processes including positive regulation of biological process, negative regulation of cellular process, negative regulation of biological process, indicating the regulation function of miRNAs, which are highly associated with the processes of chemoradiotherapy response^13^. For the CC items, the miRNA targets were enriched in the hallmarks of a cell including membrane-bounded organelle, cytosol, cytoplasm, which are critical areas with a major impact on radiation sensitivity^14^. Most GO MF items converged on the function of binding such as protein binding, macromolecular complex binding, heterocyclic compound binding, which also influence the ionizing radiation through the binding of important proteins and DNA^15^. The GO annotation results revealed the correlations between the target mRNAs of candidate miRNAs and chemoradiotherapy response.

The top enriched KEGG (Kyoto Encyclopedia of Genes and Genomes) terms revealed several pathways associated with the response to chemoradiotherapy namely pathways in cancer, microRNAs in cancer, MAPK signaling pathway, colorectal cancer, cell cycle and PI3K-Akt signaling pathway. According to the KEGG functional categories, pathways in cancer involve several important signaling pathways which have a crucial effect on proliferation, differentiation, apoptosis and invasion (cell cycle, p53 signaling pathway, TGF-β signaling pathway, MAPK signaling pathway, PI3K-Akt signaling pathway, etc). The microRNAs in cancer pathway reflects the direct relationships among these mRNAs and miRNAs in cancer^16^. MAPK signaling pathway, one of the above pathways in cancer, has been associated with the growth factor-mediated regulation of various cellular events including proliferation, differentiation and apoptosis^17^. Accumulating new evidence supports the concept that altered MAPK signaling has a high correlation with rectal carcinogenesis and radioadaptive response in DNA damage^18^. Irradiation-induced MAPK signaling causes radioresistance in cancer cells and inhibition of the MAPK signaling could render a promising therapeutic approach to intensify the sensitivity of adaptive response to radiotherapy for rectal cancer. The colorectal cancer pathway indicates that these mRNAs regulated by the candidate miRNAs play an important role in the occurrence and development of colorectal cancer including rectal cancer. The well-studied cell cycle pathway, perhaps the most vital determinant of ionizing radiation sensitivity, has been inextricably linked to the cellular response to radiotherapy in numerous studies for a long time^19^. The mechanisms responsible for cell cycle in regulating ionizing radiation have long been focused specifically on DNA damage response involved in damage recognition, cell cycle checkpoints and DNA repair^20^. Furthermore, the relative radiosensitivity of cell is determined by its cell cycle phase, with the G2-M being the most radiosensitive phase^21^. The combination of radiotherapy and intervening cell cycle by sensitizing cancer cells to ionizing radiation may provide a potential strategy to optimize the therapeutic effect^22^. Activation of the PI3K/Akt pathway has been demonstrated to play a pivotal role in cellular survival and cell cycle, contributing to the tumorigenesis and resistance to apoptosis and ionizing radiation^23^. Accumulating new evidence supports a concept that modulation of the PI3K/Akt pathway could sensitize or protect against tumor therapies in both tumor and normal tissues^24^. In a word, the evidence from pathway enrichment analyses promulgates the potential mechanism of these miRNA and their targets involved in the chemoradiotherapy response.

### PPI network construction and analysis of modules

**Figure 4:**
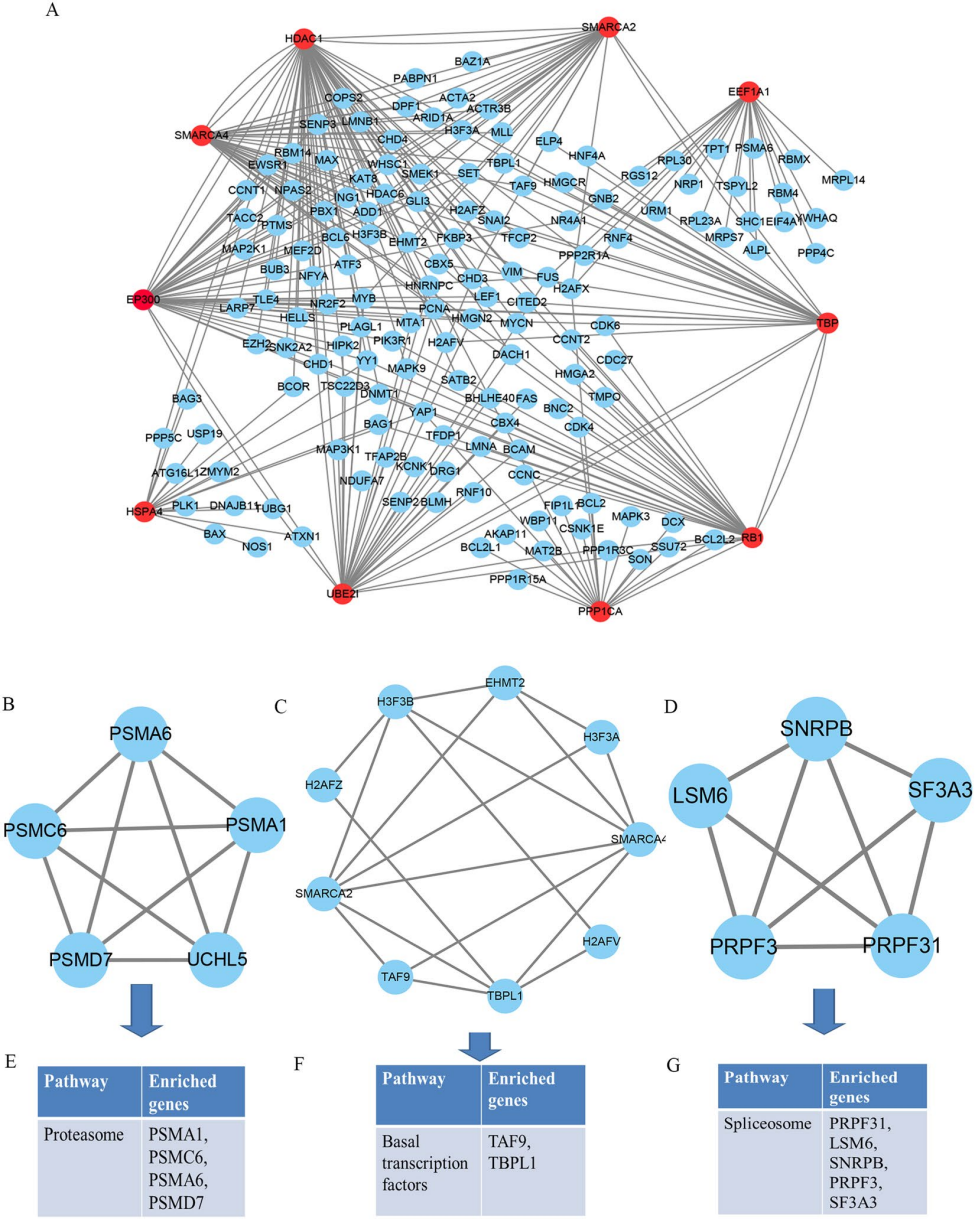
Results of PPI network analyis. (**A**) The sub-network reconstructed with the selected hub nodes and their first neighbour genes. (**B**–**D**) The top three significant modules from the PPI network. (**E**–**G**) KEGG pathway enrichment analysis for the mRNAs of the top three significant modules.

To explore the internal contact and interactions among the target mRNAs, the information received from the STRING database were integrated and constructed the PPI network. A PPI network with statistical significance made up of 751 nodes and 872 edges was detected with the set of 958 mRNAs. In the PPI network set up by the mRNAs, the average node degree was 2.32 and the top ten hub nodes with higher degrees (>18) were detected including HDAC1, SMARCA4, RB1, TBP, SMARCA2, EP300, HSPA4, UBE2I, PPP1CA and EEF1A1. The sub-network was reconstructed with the chosen hub mRNAs and their first neighbor mRNAs, outlined at Fig. 4.

We also searched the PubMed literatures for the associations of the ten mRNAs with chemoradiotherapy. Aberrant expression of HDAC1 has been found in various types of cancers, which indicates that it might be a target for cancer therapy. Increased apoptotic cell death was observed when HDAC1 was knockdown, indicating that cells were more sensitive to radiation after the inhibition of HDAC1^25^. SMARCA4 has been shown to be a novel prognostic biomarker with prediction power for the clinical survival of cisplatin-based chemotherapy in several cancers^26^. RB1 status in cancer cells has been reported to be responsible for response to radiation treatment and specific drug therapies^27^. The gene TBP, one of the housekeeping genes essential for basic maintenance of cellular processes, has been identified as an important protein involved in the response to ionizing radiation and may be used in the radiation treatment. SMARCA2 was identified involved in the early response to ionizing irradiation by cDNA microarray gene expression analysis and may be helpful for developing strategies to augment the radiation effect^28^. Recent studies have proposed that EP300 contributes to the response to radiotherapy through the involvement in cell cycle and p53 signaling^29^. Previously, HSPA4 was emerging as an important molecule in the induction of the adaptive radiation response^30^. Although the remaining three mRNAs were not reported to have direct relationships with chemoradiotherapy response, they play vital roles in rectal cancer or other cancers. For example, previous evidence supports that EEF1A1 may provide potential biomarkers for fecal RNA-based colorectal cancer screening^31^.

KEGG pathway enrichment analysis by DAVID (Database for Annotation, Visualization, and Integrated Discovery) was performed with the 10 mRNAs and the results are presented at Fig. 3. It is worth noting that three pathways consisting of viral carcinogenesis, cell cycle and Notch signaling pathway may be responsible for regulating ionizing radiation. In addition to cell cycle, another part have to mention is that the well-studied Notch signaling pathway has been a vital pathway mediating radiation resistance in tumor cells, indicating that targeting the Notch pathway may be beneficial for the synergistic improvements of radiotherapy^32^.

Using the MCODE package, the top three significant modules in the PPI network with the highest score were discovered and outlined at Fig. 4. We also conducted the enrichment analysis of the mRNAs in the top three modules by DAVID. The results indicated that these mRNAs were mostly enriched in proteasome, systemic lupus erythematosus, alcoholism, basal transcription factors and spliceosome. Proteasome is a well-studied signaling molecule related to cell survival and proliferation, the inhibitors of which may increase the sensitivity of carcinoma cells to apoptosis through elevating tumor-specific T-cell activity when combined with radiotherapy^33^. Many studies were critically reviewed the roles of transcription factors in various biological processes, such as DNA replication and repair, control of apoptosis and cellular differentiation. It is important to note that mediating the specific transcription factors may be a promising strategy to sensitize resistant tumor cells to radiotherapy^34^. Spliceosome has been proved to be closely associated with catalyzing the mRNA processing step splicing in the nucleus and its inhibitors would be promising molecular target drugs for chemotherapy^35^.

### Screening key miRNA targets and pathway analysis

Due to the large number of miRNA targets, we explored two mRNA expression profiles to investigate their expression in the microarray and identify some key mRNAs. A total of 910 and 237 DE mRNAs were obtained in GSE35452 and GSE3493 datasets, respectively. Moreover, 30 and 14 miRNA targets were identified in the DE mRNA lists retrieved from GSE35452 and GSE3493 datasets, respectively. We also carried out the literature search of the 44 mRNAs to investigate their role involved in the chemoradiotherapy response of rectal cancer (Supplementary Table S4). According to the results, most of these mRNAs correlate directly with chemoradiotherapy or rectal cancer or have contributions to the occurrence and development of other cancers, indicating their potential prediction performance. Moreover, we conducted the KEGG pathway analysis by mapping the 44 DE miRNA targets to DAVID and the results showed that they were significantly enriched in cell cycle and cGMP-PKG signaling pathway.

### Network biomarkers for predicting the response to chemoradiotherapy

**Figure 5:**
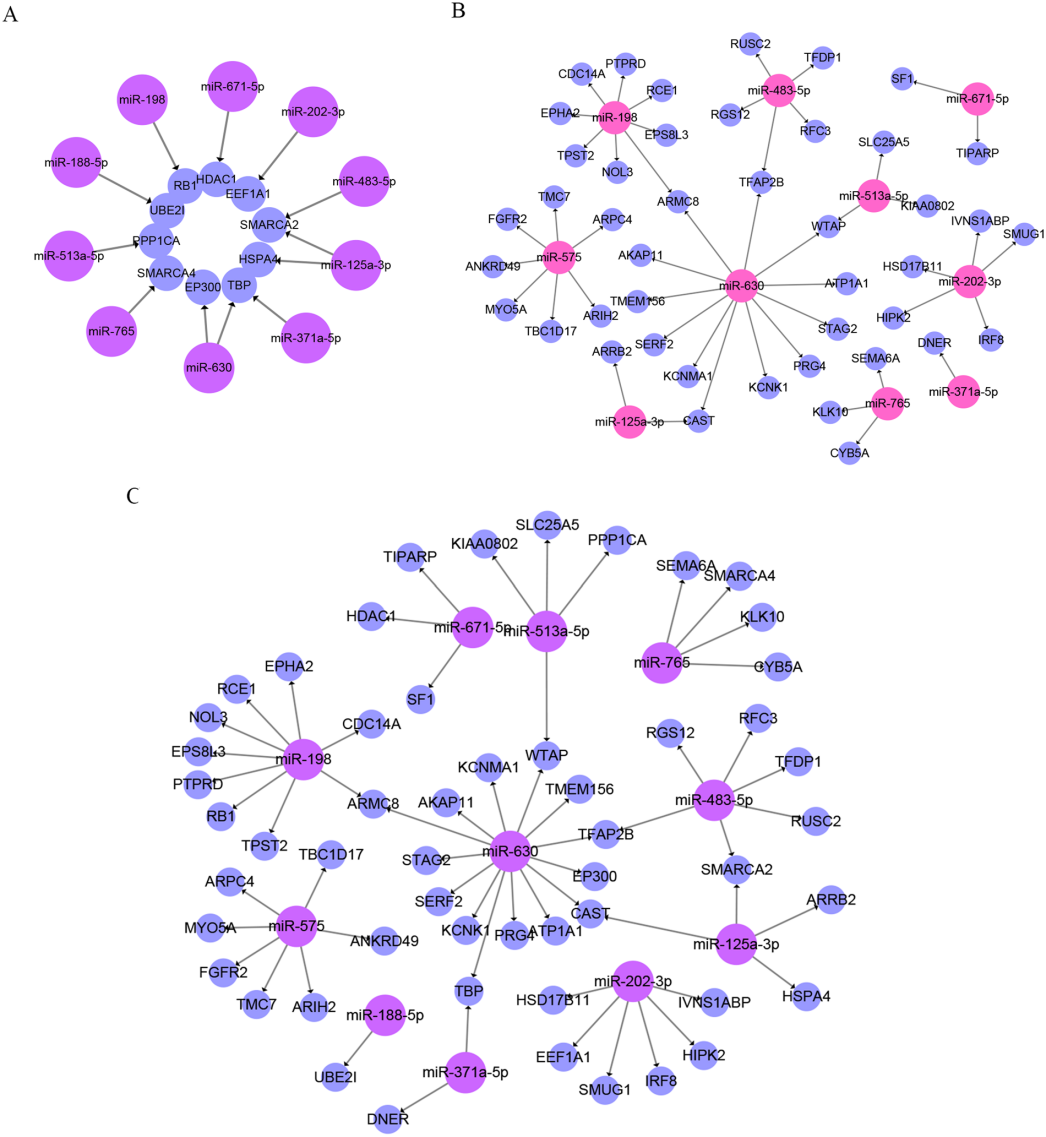
Network biomarkers for predicting the rectal cancer responses to chemoradiotherapy. (**A**) miRNA-mRNA regulatory pairs from the ten hub mRNAs and their regulated miRNAs. (**B**) miRNA-mRNA regulatory pairs from the DE mRNAs and their regulated miRNAs. (**C**). The whole network set up by the candidate miRNAs and the two lists of mRNAs.

Based on the above results, we reconstructed the ten hub mRNAs and the 44 DE miRNA targets with their regulated miRNAs and plotted them in Fig. 5A and B. Based on the above comprehensive functional analysis and validation in PubMed, the identified miRNAs and the two lists of mRNAs provide potential miRNA-mRNA regulatory pairs as network biomarkers for predicting the response to chemoradiotherapy in rectal cancer (Fig. 5C). In the combined network, eleven miRNAs along with 54 target mRNAs constructed the prediction framework including 60 regulatory pairs. One of the above candidate miRNAs (miR-622) was not found in the prediction network due to the very few target mRNAs (see Supplementary Table S1). Furthermore, superior to previous network biomarkers, the miRNA-mRNA regulatory pairs are considered to better predict the treatment response as any element in the network could have a predictive function and the prediction power may increase when they are integrated into a whole framework.

## Discussion

Many research results show that, at present, the world’s cancer treatment has entered a personalized precision treatment era. Under this background, developing tools for response prediction may help distinguish responders from non-responders to preoperative chemoradiotherapy. Patients who are not possible to respond to preoperative chemoradiotherapy will be spared from the unnecessary treatment with accompanying side effects and then subjected to alternative treatment modalities such as surgery and adjuvant therapy without delay.

An increasingly large amount of evidence has accumulated showing that miRNAs play a vital role in the chemoradiotherapy efficiency of rectal cancer. In this study, after a careful search in PubMed, a total of 43 miRNAs associated with the chemoradiotherapy response in rectal cancer were collected, of which 12 are differentially expressed in the selected miRNA expression profile. All these 12 miRNAs are supported by literature and validated by the microarray data and thus could be candidate biomarkers with high potential for predicting the chemoradiotherapy response.

We believe that if the biomarker miRNAs can predict the response of rectal cancer to preoperative chemoradiotherapy, the mRNAs they regulate should also participate in chemoradiotherapy response and diverse associated biological pathways. In order to investigate thoroughly the functions of miRNAs and their regulatory mRNAs involved in the chemoradiotherapy response, we conducted a computational functional analysis for all the targets of candidate miRNA biomarkers. Most GO terms enriched by the miRNA targets were significantly relevant to the processes of regulation at the BP level, core cell structural at CC level along with the function of binding at MF level. Furthermore, we revealed a number of novel rectal cancer related pathways from the top 15 enriched KEGG categories, such as pathways in cancer, microRNAs in cancer, MAPK signaling pathway, colorectal cancer, cell cycle and PI3K-Akt signaling pathway, which associated well with chemoradiotherapy response based on the text mining analysis. The functional enrichment analysis supported our identification of candidate miRNA biomarkers and revealed their potential mechanisms in influencing the chemoradiotherapy response. The identified pathways deserve further study for thorough mechanism.

In order to explore the internal links among the target mRNAs, we set up the PPI network based on their interaction information. Ten hub mRNAs were identified (HDAC1, SMARCA4, RB1, TBP, SMARCA2, EP300, HSPA4, UBE2I, PPP1CA and EEF1A1). Moreover, a total of 44 miRNA target mRNAs were found differentially expressed in the chosen mRNA expression datasets. We extended our search for articles related to chemoradiotherapy response of all the 54 mRNAs in PubMed. Most of the mRNAs directly participate in the chemoradiotherapy response or contribute to the initiation and progression of rectal cancer or other cancers, indicating that they also possess predictive function compared with the candidate miRNAs targeting them. All these identified mRNAs should be followed up in further experiments.

As mentioned above, we identified two lists of key target mRNAs of the candidate miRNAs from the PPI network and the mRNA expression profiles, respectively. The two lists of mRNA signatures differed considerably in terms of mRNA composition, with no overlapping mRNAs. To our surprise, however, they fall into the similar functional pathways (cell cycle) and then come to a more consistent state when enriched to systems biology levels despite the inconsistent gene lists. By convention, functionally associated mRNAs often exhibit a coordinated expression to elaborate their roles in the same functional modules, suggesting that the two lists of mRNAs may have a synergistic effect in chemoradiotherapy response^36^.

Up to now, most attention on treatment response prediction in carcinoma has been absorbed in single or limited molecules. Actually, a set of genes may be more reliable with greater power for response discrimination compared to a single gene for curative effect indications^37^. What’s more, accurate predictions are difficult as individual biomarker is hard to reveal the cancer evolutionary process at the systems biology level^38^. On the contrary, microarray analysis of mRNA or miRNA expression could make a full genome display mode at the same time, providing enormous convenience for biomarkers discovery. However, the majority of those microarray-based studies have concentrated on miRNAs or mRNAs separately. The network biomarker involving miRNA-mRNA interactions could provide novel insights in elucidating the radiation response process at the molecular level^39^. Cancer is a highly heterogeneous disease; consequently, the future exploration of cancer biomarker should be rooted in systematical and dynamical manner. The network-based approach of the present study identified potential biomarkers for predicting chemoradiotherapy efficiency and provided a systemic method to integrate diverse information into a systematical framework. In this study, the candidate miRNAs, along with their target mRNAs, provide potential miRNA-mRNA regulatory pairs as network biomarkers for predicting the chemoradiotherapy response. To validate the accuracy of our predictive results, although not perfect, we have performed integrative and comprehensive bioinformatics analysis of them and evaluated them through the literature verification. Compared to previous network biomarkers, our miRNA-mRNA regulatory pairs are considered to be better predictors of therapeutic response since any element in the network has predictive ability. It is worth noting that the predictive power may increase when the whole network nodes are integrated into a complete frame. As a future perspective, edge-variation in the network may be considered for the further improvement of the therapeutic response and biological experiments are still necessary for further validation.

Nowadays, many efforts have been paid in attempt to solve the troublesome problems in clinical and some effective computational models for miRNA-disease associations have been developed^40–44^. These tools hold great promise for providing precision medicine and would be important biological resources for experimental guidance. Nevertheless, all biomarkers, including genomic and molecular tools must have analytic validity, clinical validity, and clinical utility prior to incorporation into clinical care. Once the computational model is analytically valid, it must be shown in multiple independent cohorts to have the ability to be accurate and reproducible. However, promising achievement that could extend to clinical application remains rare. Clinically useful cancer biomarkers remain limited as cancer is a complex disease. The complexity of cancer could be more clearly comprehended by the cancer hallmarks which are important for cancer biomarker discovery. In our prediction model, some enriched terms are associated with cancer hallmarks (abnormal metabolic pathways, genome instability, cell growth, death, etc), which may be helpful for the explanation of the prediction power^45, 46^. In addition to provide the prediction model, our study also provides potential therapeutic strategies for tumor cell radiosensitization when elucidating the underlying mechanism influencing the chemoradiotherapy response. For example, by intervening the key miRNAs or mRNAs involved in the identified pathways may help enhance the chemoradiotherapy sensitivity^47^. Of course, the relevant researches need to continue.

In summary, we applied an integrative approach to identify miRNA-mRNA target pairs as network biomarkers for predicting the response of rectal cancer to chemoradiotherapy. The constructed network biomarkers not only have a strong predictive function but also provide systematic insights into the mechanisms underlying the therapeutic outcome difference which could be prioritized for further biological experiments.

## Materials and Methods

### Data collection

The miRNA expression profile (GSE29298)^48^ and mRNA expression datasets (GSE35452, GSE3493)^49, 50^ were retrieved from the Gene Expression Omnibus (GEO) based on the public National Center for Biotechnology Information (NCBI) database. Table 2 gives detailed information of the miRNA and mRNA expression datasets. All the datasets were downloaded in normalized data file format for further analysis. The potential miRNAs associated with the response to chemoradiotherapy were searched in PubMed and only the miRNA validated in biological experiment or identified through predictive methods were collected.

**Table 2:** Summary of microarray datasets used in this study.

GEO Accession	PMID	Platform	Probe Number	Number of samples	
				Responders	Non-responders
GSE29298	22172905	Agilent-021827 Human miRNA Microarray	796	9	29
GSE35452	24316942	Affymetrix Human Genome U133 Plus 2.0 Array	54675	24	22
GSE3493	16585155	Affymetrix Human Genome U95 Version 2 Array	12626	11	35

### Differential expression analysis

The miRNAs and mRNAs differentially expressed (DE) between the responders and non-responders to preoperative chemoradiotherapy in rectal cancer were extracted based on Student’s t test in the Limma R package^51^. A p-value < 0.05 was selected as the cutoff value of statistical significance.

### Targets of miRNA prediction

The mRNAs targeted by the candidate miRNAs were obtained by combining four experimentally confirmed interactions (miRecords^52^, miR2Disease^53^, miRTarbase^54^ and Tarbase^55^) with three computationally predicted data (ExprTargetDB^56^, HOCTAR^57^ and starBase^58^). For the miRNA-mRNA target pairs with experimental evidence, the regulation data was directly adopted while for the computational prediction data, miRNA-mRNA regulatory interactions residing in no fewer than two databases from these three target prediction algorithms were selected.

### Gene ontology and pathway enrichment analysis

GO and pathway analysis were conducted with all the target mRNAs of candidate miRNAs on the basis of the STRING database^59^. For the overlapped mRNAs between miRNA targets and DE mRNAs, the KEGG enrichment pathway analysis was carried out using the DAVID tool^60^. P-value < 0.05 and count ≥2 were considered as the cut-off criteria. The enriched results were evaluated by a thorough search in PubMed for the supporting documents.

### PPI network construction and analysis

The target mRNAs regulated by the candidate miRNAs were mapped to the STRING database to evaluate the PPI information. The PPI data validated by biological experiments with the combined score >0.4 were selected and then visualized with the powerful tool Cytoscape^61^. The PPI network analysis returned the key hub mRNAs with high degrees in the constructed network and the significant modules were identified with the plug-in Molecular Complex Detection (MCODE) of Cytoscape. Then KEGG pathway analysis was performed with the chosen hub mRNAs by using DAVID. P value < 0.05 was regarded as statistically significant differences.

## Electronic supplementary material

Supplementary Information
