# Supplementary material for: Screening candidate microRNA-mRNA regulatory pairs for predicting the response to chemoradiotherapy in rectal cancer by a bioinformatics approach
Source: Sci Rep. 2017 Sep 12;7:11312. doi: 10.1038/s41598-017-11840-7 (PMC5595906; doi:10.1038/s41598-017-11840-7)
Supplement: Supplementary file 1 — Supplementary Information [file 41598_2017_11840_MOESM1_ESM.pdf]

Screening candidate microRNA-mRNA regulatory pairs for predicting the response to chemoradiotherapy in rectal cancer by a bioinformatics approach

Qiliang Peng[1, 2, 3, 4, *], Junjia Zhu[5, 6, *], Peipei Shen[1, 2, 3, 4], Wenyan Yao[1, 2, 3, 4], Yu Lei[1, 2, 3, 4], Li Zou[1, 2, 3, 4], Yingying Xu[1, 2, 3, 4], Yuntian Shen[1, 2, 3, 4], Yaqun Zhu[1, 2, 3, 4]

[1]Department of Radiotherapy & Oncology, The Second Affiliated Hospital of Soochow University, Suzhou, China

[2]Institute of Radiotherapy & Oncology, Soochow University, Suzhou, China

[3]Suzhou Key Laboratory for Radiation Oncology, Suzhou, China

[4]Suzhou Medical Center of Radiotherapy & Oncology, Suzhou, China

[5]Department of Anorectal Surgery, The Affiliated Jiangyin Hospital of Southeast University Medical College, Jiangyin, China

[6]Department of General Surgery, The Second Affiliated Hospital of Soochow University, Suzhou, China.

*These authors contributed equally to this work.

Correspondence to: Yaqun Zhu, Dept. of Radiotherapy & Oncology, the Second Affiliated Hospital of Soochow University, San Xiang Road No. 1055, Suzhou, Jiangsu 215004, China

Phone: +86-512-67784826

Fax: +86-512-68284303

Email: szzhuyaqun@sina.com

Supplementary Information

Table S1: The miRNA-mRNA regulatory pairs.

miRNA ID	mRNA ID
miR-630	KIAA1324
miR-630	MEF2D
miR-630	EP300
miR-630	BHLHB2
miR-630	VIL2
miR-630	SOCS2
miR-630	ALDOB
miR-630	TEX264
miR-630	KCNMA1
miR-630	PTCD2
miR-630	TJP1
miR-630	HSPB8
miR-630	FKBP3
miR-630	GSTT2
miR-630	KCNK1
miR-630	NPAS2
miR-630	RBMX
miR-630	RBM4
miR-630	MSMB
miR-630	VPS35
miR-630	HDAC6
miR-630	SULT2A1
miR-630	ZMYM2
miR-630	KALRN
miR-630	ATP6V0C
miR-630	YIPF6
miR-630	PIK3R1
miR-630	SMEK1
miR-630	MAP3K2
miR-630	TAC3
miR-630	FKBP1B
miR-630	PRDM13
miR-630	TFAP2B
miR-630	PSMA1
miR-630	SPRY1
miR-630	SERF2
miR-630	TAGLN3
miR-630	ATP1A1
miR-630	BCOR
miR-630	BCL2

(Continued)

miRNA ID	mRNA ID
miR-630	BBOX1
miR-630	MBNL2
miR-630	CENTG2
miR-630	SNTG2
miR-630	SNTB2
miR-630	KRT24
miR-630	KRT83
miR-630	UTP6
miR-630	SEMA3D
miR-630	SEMA7A
miR-630	TMEM156
miR-630	ARMC8
miR-630	ARL8B
miR-630	PPP2R5B
miR-630	AKAP11
miR-630	TFG
miR-630	KIAA0146
miR-630	EZH2
miR-630	PPP1R3C
miR-630	TBP
miR-630	CASQ1
miR-630	CSAD
miR-630	CRISP3
miR-630	PCDH11X
miR-630	YTHDF1
miR-630	ASPA
miR-630	WTAP
miR-630	NMU
miR-630	LRP6
miR-630	ARF4
miR-630	GNL3L
miR-630	CLPS
miR-630	HIST1H2AE
miR-630	TIPRL
miR-630	VTCN1
miR-630	CLK2
miR-630	PRG4
miR-630	CACNA1G
miR-630	LAPTM4A
miR-630	SEC61G

(Continued)

miRNA ID	mRNA ID
miR-630	CDC27
miR-630	SNAI2
miR-630	GALNT1
miR-630	SENP3
miR-630	LMNB1
miR-630	COMMD10
miR-630	PUM1
miR-630	VIM
miR-630	KIF2A
miR-630	USP34
miR-630	ANKHD1
miR-630	CA2
miR-630	SPINK5
miR-630	CBX5
miR-630	CAST
miR-630	YAP1
miR-630	HMGCR
miR-630	BCL2L2
miR-630	DNAJC13
miR-630	STAG2
miR-630	TOMM7
miR-630	FAM12B
miR-630	PLK1
miR-630	NEO1
miR-630	FCER1A
miR-630	TDRKH
miR-630	PAWR
miR-630	ZNF187
miR-630	KDR
miR-630	PAEP
miR-630	ZNF410
miR-630	ZNF384
miR-630	IL10
miR-630	GIPC1
miR-630	FAM98A
miR-630	ZNF536
miR-630	PDCL
miR-630	DCP1A
miR-630	ERICH1
miR-630	CEP192

(Continued)

miRNA ID	mRNA ID
miR-630	ZNF711
miR-202-3p	ZMYM2
miR-202-3p	SMAP1
miR-202-3p	SSU72
miR-202-3p	TRIM71
miR-202-3p	NGLY1
miR-202-3p	PVRL3
miR-202-3p	SNX5
miR-202-3p	ZNF193
miR-202-3p	ISG20L2
miR-202-3p	HPCAL1
miR-202-3p	CEP55
miR-202-3p	HSD17B11
miR-202-3p	C1orf43
miR-202-3p	SRGN
miR-202-3p	FKRP
miR-202-3p	LARP7
miR-202-3p	RPL23A
miR-202-3p	TCOF1
miR-202-3p	SLMAP
miR-202-3p	H2AFZ
miR-202-3p	MTHFD2
miR-202-3p	GABBR1
miR-202-3p	TAF9
miR-202-3p	MTMR4
miR-202-3p	SULF2
miR-202-3p	KLF12
miR-202-3p	SMNDC1
miR-202-3p	DDX50
miR-202-3p	HIGD2A
miR-202-3p	MON2
miR-202-3p	POLR3D
miR-202-3p	DNAJB11
miR-202-3p	ATXN10
miR-202-3p	RBM9
miR-202-3p	RBM4
miR-202-3p	CHPT1
miR-202-3p	PBX3
miR-202-3p	EEF1A1
miR-202-3p	PANX2

(Continued)

miRNA ID	mRNA ID
miR-202-3p	ZSWIM4
miR-202-3p	TRIM5
miR-202-3p	FUS
miR-202-3p	MYCN
miR-202-3p	IRF8
miR-202-3p	C21orf63
miR-202-3p	DBT
miR-202-3p	ZBTB10
miR-202-3p	RNPS1
miR-202-3p	PCNA
miR-202-3p	C18orf21
miR-202-3p	SLC31A2
miR-202-3p	EDC4
miR-202-3p	SENP2
miR-202-3p	NR2F2
miR-202-3p	TMEM138
miR-202-3p	SF3A3
miR-202-3p	GTPBP2
miR-202-3p	CKAP5
miR-202-3p	HNRPC
miR-202-3p	POLQ
miR-202-3p	VGLL3
miR-202-3p	BBS10
miR-202-3p	LRRC41
miR-202-3p	IVNS1ABP
miR-202-3p	TEX261
miR-202-3p	PYGO2
miR-202-3p	ARMCX5
miR-202-3p	CANT1
miR-202-3p	KLHDC3
miR-202-3p	SMUG1
miR-202-3p	HIPK2
miR-202-3p	SPCS1
miR-202-3p	MAP3K1
miR-202-3p	HTATIP2
miR-202-3p	DNAJC1
miR-202-3p	KLHL15
miR-202-3p	ASXL1
miR-202-3p	RAB2B
miR-202-3p	LSM6

(Continued)

miRNA ID	mRNA ID
miR-202-3p	RNF13
miR-202-3p	C3orf63
miR-202-3p	SLC39A8
miR-575	SACS
miR-575	MS4A4A
miR-575	RAB6IP1
miR-575	EHMT2
miR-575	NR4A1
miR-575	PLAGL1
miR-575	PRKAG2
miR-575	RPS6KA1
miR-575	RPS6KA3
miR-575	PIK3CD
miR-575	RAB38
miR-575	CACNA2D1
miR-575	FKBP14
miR-575	GPR63
miR-575	TIPRL
miR-575	MAP2K1
miR-575	WDR57
miR-575	NRP1
miR-575	SLC14A1
miR-575	CHD3
miR-575	RLF
miR-575	KIAA1794
miR-575	CHRD
miR-575	ENPP2
miR-575	FGFR2
miR-575	TMC7
miR-575	TMEM4
miR-575	RGS3
miR-575	PPP1R15A
miR-575	UBAP2
miR-575	FAS
miR-575	CCNT2
miR-575	DCHS1
miR-575	TPT1
miR-575	HIF3A
miR-575	ANKRD46
miR-575	ANKRD49

(Continued)

miRNA ID	mRNA ID
miR-575	SPINK5
miR-575	NDUFB1
miR-575	RBM14
miR-575	HMGA2
miR-575	MYO10
miR-575	TACSTD1
miR-575	STOML1
miR-575	MYO5A
miR-575	USP19
miR-575	VASP
miR-575	MID2
miR-575	IFNGR2
miR-575	DPP8
miR-575	KCTD13
miR-575	TBC1D17
miR-575	MRPS7
miR-575	COX6B1
miR-575	GALNT1
miR-575	POLD4
miR-575	ARPC4
miR-575	LMBRD1
miR-575	COPS2
miR-575	SNRPB
miR-575	FGF1
miR-575	ARIH2
miR-575	TUBG1
miR-575	UCHL5
miR-575	ACTR3B
miR-765	DTX2
miR-765	PSMA6
miR-765	IGSF1
miR-765	PFKFB3
miR-765	MAP3K4
miR-765	SLC25A37
miR-765	TACC2
miR-765	PDE1C
miR-765	ST3GAL2
miR-765	HNF4A
miR-765	GNL3L
miR-765	SV2A

(Continued)

miRNA ID	mRNA ID
miR-765	BFSP2
miR-765	BCL6
miR-765	STK4
miR-765	BAG1
miR-765	TAOK3
miR-765	BAX
miR-765	NRBP1
miR-765	SCAMP3
miR-765	CFP
miR-765	IQCB1
miR-765	FAM117A
miR-765	KLK10
miR-765	IGFBP2
miR-765	TAGLN
miR-765	DNAJC7
miR-765	LIN7B
miR-765	TUBG1
miR-765	MGA
miR-765	SGCE
miR-765	FKBP1A
miR-765	LBP
miR-765	APOB
miR-765	FUS
miR-765	PIB5PA
miR-765	COLEC12
miR-765	LMNA
miR-765	LMO1
miR-765	EZH2
miR-765	CENPT
miR-765	NECAP2
miR-765	CSH2
miR-765	JARID1C
miR-765	MYO1A
miR-765	MYO1C
miR-765	USP19
miR-765	GAPVD1
miR-765	PUM1
miR-765	EIF4G1
miR-765	NRXN2
miR-765	NYX

(Continued)

miRNA ID	mRNA ID
miR-765	BEXL1
miR-765	EIF2C1
miR-765	EPHB2
miR-765	PREB
miR-765	SPINK5
miR-765	NTRK3
miR-765	ADIPOR1
miR-765	PTGDS
miR-765	CYB5A
miR-765	NEU3
miR-765	SPTLC3
miR-765	GPC3
miR-765	NDUFA7
miR-765	SMARCA4
miR-765	ACTA2
miR-765	TRAF3IP2
miR-765	PVRL1
miR-765	SH2D2A
miR-765	WHSC1
miR-765	PCMT1
miR-765	CACNA1G
miR-765	XPNPEP1
miR-765	TPM3
miR-765	AGPAT7
miR-765	HTRA1
miR-765	RGS7
miR-765	SEMA6A
miR-765	PRPF3
miR-765	OSBPL7
miR-765	TLE4
miR-765	HOXC6
miR-765	HOXB8
miR-765	KIRREL
miR-765	HIF3A
miR-765	PRR13
miR-765	RBM4
miR-765	RALY
miR-765	BST1
miR-765	TIAL1
miR-765	CAPN3

(Continued)

miRNA ID	mRNA ID
miR-765	CCDC51
miR-765	PPP4C
miR-765	PPP5C
miR-765	IDH1
miR-765	COX6B1
miR-765	TMEM160
miR-513a-5p	LITAF
miR-513a-5p	HOXD11
miR-513a-5p	MRPL14
miR-513a-5p	CLK2
miR-513a-5p	KIAA0802
miR-513a-5p	FYN
miR-513a-5p	FAM107B
miR-513a-5p	WTAP
miR-513a-5p	B7-H
miR-513a-5p	MBD6
miR-513a-5p	MBIP
miR-513a-5p	CD274
miR-513a-5p	TXNRD1
miR-513a-5p	APCDD1
miR-513a-5p	MIA3
miR-513a-5p	EWSR1
miR-513a-5p	HELLS
miR-513a-5p	MND1
miR-513a-5p	SLC25A5
miR-513a-5p	PPP1CA
miR-513a-5p	SERPINH1
miR-513a-5p	MAPKAPK3
miR-513a-5p	STMN1
miR-198	RAP1GAP
miR-198	NUMA1
miR-198	COL5A1
miR-198	EPS8L3
miR-198	BCL2L1
miR-198	FAT
miR-198	COL6A3
miR-198	BUB3
miR-198	FUS
miR-198	TARBP2
miR-198	MARCH2

(Continued)

miRNA ID	mRNA ID
miR-198	BNC1
miR-198	SLC25A28
miR-198	DCX
miR-198	EPB41L1
miR-198	RGS10
miR-198	H3F3A
miR-198	CLU
miR-198	TCTA
miR-198	PIK3C2G
miR-198	TIMP1
miR-198	RB1
miR-198	CNOT3
miR-198	CD160
miR-198	CCNT1
miR-198	DPF1
miR-198	EPHA2
miR-198	PAPPA
miR-198	ARHGAP19
miR-198	MLL2
miR-198	FBXL5
miR-198	MYB
miR-198	SEMA7A
miR-198	TITF1
miR-198	DENND2D
miR-198	HOOK2
miR-198	LBP
miR-198	CDC14A
miR-198	CENPE
miR-198	VAT1
miR-198	SLC8A1
miR-198	TXNDC9
miR-198	PIGC
miR-198	NR2F6
miR-198	CTNNA3
miR-198	CD84
miR-198	FIP1L1
miR-198	CDK4
miR-198	CDK6
miR-198	FAM62A
miR-198	SPRY2

(Continued)

miRNA ID	mRNA ID
miR-198	PSMD7
miR-198	PSME3
miR-198	PLAU
miR-198	RRAS
miR-198	MBNL2
miR-198	SLC35A2
miR-198	NFYA
miR-198	FAM46A
miR-198	SLC16A1
miR-198	GNB2
miR-198	GOLGB1
miR-198	CALU
miR-198	NDRG3
miR-198	PHKB
miR-198	GNL1
miR-198	FBLN2
miR-198	PHF8
miR-198	RNF4
miR-198	PAK2
miR-198	LIN7B
miR-198	TPST2
miR-198	ANG
miR-198	TMPO
miR-198	CSF2RA
miR-198	PBX1
miR-198	SAPS1
miR-198	CKMT1A
miR-198	CELSR2
miR-198	SNX17
miR-198	RCE1
miR-198	RFXANK
miR-198	ATXN1
miR-198	KRT76
miR-198	SEC24C
miR-198	SEC24B
miR-198	IL10
miR-198	TLE4
miR-198	TCF7L1
miR-198	CTSC
miR-198	CTNNBL1

(Continued)

miRNA ID	mRNA ID
miR-198	APH1A
miR-198	APEH
miR-198	ZNF281
miR-198	ZNF289
miR-198	IL11RA
miR-198	ARMC8
miR-198	HIVEP2
miR-198	PCDH12
miR-198	PLXNA1
miR-198	VPS35
miR-198	NCKAP1L
miR-198	IFNGR2
miR-198	EIF4E2
miR-198	ARID1A
miR-198	NDUFC1
miR-198	GAPVD1
miR-198	ALPL
miR-198	TFCP2
miR-198	UFC1
miR-198	GPRC5C
miR-198	ZCCHC4
miR-198	H2AFX
miR-198	NTRK3
miR-198	AIM1L
miR-198	CLTA
miR-198	CRISP3
miR-198	GZMH
miR-198	JAM2
miR-198	PGAP1
miR-198	PTMS
miR-198	SMC1A
miR-198	NOL3
miR-198	SLITRK5
miR-198	CHD1
miR-198	CHD4
miR-198	ACHE
miR-198	ACE2
miR-198	ATG9A
miR-198	HPRT1
miR-198	ATG16L1

(Continued)

miRNA ID	mRNA ID
miR-198	DHRS1
miR-198	PPA2
miR-198	SRGAP2
miR-198	RBM12
miR-198	ADH5
miR-198	RBM4B
miR-198	WNT7A
miR-198	BAI3
miR-198	CXCR7
miR-198	MAP4
miR-198	HABP2
miR-198	c-Myb
miR-198	USP16
miR-198	PTPRD
miR-198	BCL9
miR-198	WAC
miR-198	SH3GLB1
miR-198	LASS4
miR-198	TAZ
miR-198	STAU2
miR-198	DDR2
miR-198	SCD
miR-198	PPP2CB
miR-198	DFNB31
miR-198	PCDH1
miR-198	UBE2M
miR-198	PKD2L2
miR-198	URM1
miR-198	ITGB4BP
miR-198	RIMS3
miR-198	CDK2AP2
miR-198	PLAUR
miR-198	NXF1
miR-198	ARAF
miR-198	ARL6IP1
miR-198	SGSH
miR-198	FABP5
miR-198	SHC1
miR-198	ZDHHC6
miR-198	DCTN3

(Continued)

miRNA ID	mRNA ID
miR-198	TBPL1
miR-198	M6PR
miR-198	CXCL13
miR-483-5p	RUSC2
miR-483-5p	FBN2
miR-483-5p	NRBP1
miR-483-5p	SILV
miR-483-5p	ARHGEF9
miR-483-5p	ZNF289
miR-483-5p	PPP2R1A
miR-483-5p	SHOX2
miR-483-5p	ALDOC
miR-483-5p	TFDP1
miR-483-5p	PABPN1
miR-483-5p	LYPLA2
miR-483-5p	VAMP8
miR-483-5p	FHL1
miR-483-5p	DBN1
miR-483-5p	CDKL3
miR-483-5p	PREP
miR-483-5p	B4GALT3
miR-483-5p	RBM35A
miR-483-5p	SAMD4A
miR-483-5p	JARID1C
miR-483-5p	TFAP2B
miR-483-5p	CHD1
miR-483-5p	NFX1
miR-483-5p	SMARCA2
miR-483-5p	UBE2D2
miR-483-5p	H2AFV
miR-483-5p	CTSK
miR-483-5p	SH3GL3
miR-483-5p	PDGFD
miR-483-5p	APBB2
miR-483-5p	ZCWPW1
miR-483-5p	CACNB1
miR-483-5p	KLHDC3
miR-483-5p	MRPL52
miR-483-5p	UCK2
miR-483-5p	DNAJC7

(Continued)

miRNA ID	mRNA ID
miR-483-5p	PIP5K1A
miR-483-5p	RFC3
miR-483-5p	MTHFD2L
miR-483-5p	MAN1A2
miR-483-5p	GCC2
miR-483-5p	SLC25A15
miR-483-5p	RBM5
miR-483-5p	RGS12
miR-483-5p	RNF10
miR-483-5p	GNLY
miR-483-5p	NAB2
miR-483-5p	CD53
miR-483-5p	AP2M1
miR-483-5p	RHOA
miR-483-5p	MON1B
miR-483-5p	ADRBK1
miR-483-5p	FOXJ2
miR-483-5p	SLC23A2
miR-483-5p	MYO9B
miR-483-5p	USP48
miR-483-5p	BCAM
miR-483-5p	BAG1
miR-483-5p	MEA1
miR-483-5p	CITED2
miR-483-5p	PDE4DIP
miR-483-5p	PRIM2A
miR-483-5p	MLL
miR-483-5p	MAX
miR-483-5p	MAPK3
miR-483-5p	CD320
miR-483-5p	SON
miR-483-5p	SET
miR-483-5p	UBE2Z
miR-483-5p	DNMT1
miR-483-5p	CAMK2G
miR-483-5p	DRG1
miR-483-5p	ISGF3G
miR-371a-5p	BAG3
miR-371a-5p	ZNF564
miR-371a-5p	ZNF503

(Continued)

miRNA ID	mRNA ID
miR-371a-5p	TBP
miR-371a-5p	ZNF644
miR-371a-5p	HTRA1
miR-371a-5p	SPTBN1
miR-371a-5p	SEC23A
miR-371a-5p	SLC35A5
miR-371a-5p	KIAA0907
miR-371a-5p	PSMC6
miR-371a-5p	ENOPH1
miR-371a-5p	BLMH
miR-371a-5p	MLL2
miR-371a-5p	C9orf23
miR-371a-5p	WBP11
miR-371a-5p	CAMK2G
miR-371a-5p	DNER
miR-371a-5p	MAT2B
miR-371a-5p	ZNHIT3
miR-371a-5p	GEMIN5
miR-371a-5p	VPS37C
miR-371a-5p	HBS1L
miR-371a-5p	BTG3
miR-371a-5p	CNOT3
miR-371a-5p	CITED2
miR-371a-5p	ITGB8
miR-371a-5p	BAZ1A
miR-371a-5p	DCP1A
miR-371a-5p	MYCN
miR-371a-5p	TLE4
miR-371a-5p	CCNC
miR-371a-5p	ZDHHC13
miR-371a-5p	ZDHHC23
miR-371a-5p	CBX4
miR-371a-5p	TUBB
miR-371a-5p	PHF2
miR-371a-5p	TTF1
miR-371a-5p	CALU
miR-371a-5p	NME4
miR-371a-5p	NDFIP1
miR-371a-5p	HMGN2
miR-371a-5p	AAMP

(Continued)

miRNA ID	mRNA ID
miR-371a-5p	NGRN
miR-371a-5p	CKS2
miR-371a-5p	LEF1
miR-371a-5p	EPN1
miR-371a-5p	GHITM
miR-371a-5p	NUF2
miR-371a-5p	GSR
miR-371a-5p	GLRX2
miR-371a-5p	LRRC42
miR-371a-5p	COPS4
miR-371a-5p	CSNK1E
miR-371a-5p	CR2
miR-371a-5p	YTHDF1
miR-371a-5p	SIPA1L2
miR-371a-5p	TTC21B
miR-371a-5p	GOLGA7
miR-371a-5p	BECN1
miR-371a-5p	INTS3
miR-371a-5p	CTNNA1
miR-371a-5p	UNKL
miR-671-5p	SELP
miR-671-5p	LRP8
miR-671-5p	PIP5K2C
miR-671-5p	ESRRA
miR-671-5p	PIP5K3
miR-671-5p	ATF3
miR-671-5p	SEPT6
miR-671-5p	HLA-DRB1
miR-671-5p	PRPF31
miR-671-5p	GPR125
miR-671-5p	FGL1
miR-671-5p	SCMH1
miR-671-5p	NOS1
miR-671-5p	SPTBN1
miR-671-5p	SPTBN2
miR-671-5p	AKT2
miR-671-5p	SATB2
miR-671-5p	DNAJB5
miR-671-5p	BTNL8
miR-671-5p	HCN2

(Continued)

miRNA ID	mRNA ID
miR-671-5p	COX5A
miR-671-5p	UBAP2L
miR-671-5p	SSBP1
miR-671-5p	RNF38
miR-671-5p	RAB13
miR-671-5p	ADD1
miR-671-5p	TSPYL2
miR-671-5p	DCTN2
miR-671-5p	PSG1
miR-671-5p	EIF2B3
miR-671-5p	CD34
miR-671-5p	PHF15
miR-671-5p	PKN2
miR-671-5p	ELP4
miR-671-5p	MYST1
miR-671-5p	TIPARP
miR-671-5p	SF1
miR-671-5p	CLINT1
miR-671-5p	CLEC4E
miR-671-5p	YWHAQ
miR-671-5p	IL10
miR-671-5p	BSDC1
miR-671-5p	RAP1GDS1
miR-671-5p	LYZL6
miR-671-5p	MYL6
miR-671-5p	MAX
miR-671-5p	VSIG4
miR-671-5p	CAPN6
miR-671-5p	MATN4
miR-671-5p	CSF3R
miR-671-5p	DVL3
miR-671-5p	ANKRD25
miR-671-5p	KLF6
miR-671-5p	CSNK2A2
miR-671-5p	IGF2
miR-671-5p	KCNQ3
miR-671-5p	COL16A1
miR-671-5p	HSPH1
miR-671-5p	IL17RB
miR-671-5p	HDAC1

(Continued)

miRNA ID	mRNA ID
miR-671-5p	HMGA2
miR-671-5p	SPTB
miR-671-5p	HRH3
miR-671-5p	GON4L
miR-671-5p	GFRA1
miR-671-5p	RHOBTB2
miR-671-5p	TRIP13
miR-671-5p	PTGDS
miR-125a-3p	CCDC28B
miR-125a-3p	CLPTM1
miR-125a-3p	TSC22D3
miR-125a-3p	FLNA
miR-125a-3p	KLHL20
miR-125a-3p	CDC123
miR-125a-3p	HSPA4
miR-125a-3p	ZNF3
miR-125a-3p	YY1
miR-125a-3p	ARRB2
miR-125a-3p	DDX54
miR-125a-3p	BMP4
miR-125a-3p	BNC2
miR-125a-3p	C21orf63
miR-125a-3p	MRRF
miR-125a-3p	MTA1
miR-125a-3p	C9orf89
miR-125a-3p	MLLT10
miR-125a-3p	GIT1
miR-125a-3p	MORN2
miR-125a-3p	TPT1
miR-125a-3p	CAST
miR-125a-3p	CALR
miR-125a-3p	GLI3
miR-125a-3p	NCLN
miR-125a-3p	TRIP12
miR-125a-3p	SIPA1L1
miR-125a-3p	RPL13A
miR-125a-3p	RPL30
miR-125a-3p	CLK2
miR-125a-3p	SMARCA2
miR-125a-3p	UBL5
miR-125a-3p	FOXC1

(Continued)

miRNA ID	mRNA ID
miR-125a-3p	ZDHHC6
miR-125a-3p	MAPK9
miR-125a-3p	DACH1
miR-125a-3p	IGJ
miR-188-5p	UBE2I
miR-188-5p	H3F3B
miR-188-5p	SIPA1L2
miR-188-5p	CNIH
miR-188-5p	SERTAD3
miR-188-5p	ARPC2
miR-188-5p	EIF4A1
miR-188-5p	PLD3
miR-188-5p	EIF1
miR-188-5p	SUMF1
miR-188-5p	PAFAH1B1
miR-188-5p	C9orf6
miR-188-5p	RAB14
miR-188-5p	MCAT
miR-188-5p	B4GALT3
miR-622	ING1
miR-622	KRAS
miR-622	K-Ras

Table S2: The significantly enriched KEGG pathways by targets of candidate miRNA biomarkers.

Pathway ID	Pathway description	Gene count	False discovery rate
4520	Adherens junction	13	1.30E-4
5200	Pathways in cancer	31	1.30E-4
5206	MicroRNAs in cancer	19	1.30E-4
4010	MAPK signaling pathway	25	4.64E-4
5210	Colorectal cancer	10	1.90E-3
5220	Chronic myeloid leukemia	11	1.90E-3
5166	HTLV-I infection	23	2.15E-3
5213	Endometrial cancer	9	2.15E-3
5214	Glioma	10	2.15E-3
5215	Prostate cancer	12	2.15E-3
4110	Cell cycle	14	2.94E-3
4114	Oocyte meiosis	13	2.94E-3
4151	PI3K-Akt signaling pathway	27	2.94E-3
5223	Non-small cell lung cancer	9	2.94E-3
5218	Melanoma	10	2.94E-3
4810	Regulation of actin cytoskeleton	19	4.52E-3
4141	Protein processing in endoplasmic reticulum	16	5.06E-3
4722	Neurotrophin signaling pathway	13	5.06E-3
5202	Transcriptional misregulation in cancer	16	5.23E-3
5212	Pancreatic cancer	9	5.23E-3
4152	AMPK signaling pathway	13	5.93E-3
5205	Proteoglycans in cancer	19	5.93E-3
4360	Axon guidance	13	7.15E-3
5161	Hepatitis B	14	7.15E-3
5169	Epstein-Barr virus infection	17	7.15E-3
4261	Adrenergic signaling in cardiomyocytes	14	7.71E-3
4390	Hippo signaling pathway	14	1.05E-2
4510	Focal adhesion	17	1.34E-2
4066	HIF-1 signaling pathway	11	1.35E-2
5412	Arrhythmogenic right ventricular cardiomyopathy (ARVC)	9	1.35E-2
5152	Tuberculosis	15	1.43E-2
5034	Alcoholism	13	1.45E-2
4144	Endocytosis	16	1.72E-2
4720	Long-term potentiation	8	1.75E-2
4914	Progesterone-mediated oocyte maturation	9	1.96E-2
4530	Tight junction	12	1.99E-2
5216	Thyroid cancer	5	2.10E-2
5203	Viral carcinogenesis	15	2.15E-2

4910	Insulin signaling pathway	12	2.53E-2
5222	Small cell lung cancer	9	2.61E-2
5221	Acute myeloid leukemia	7	2.68E-2
3015	mRNA surveillance pathway	9	2.92E-2
4630	Jak-STAT signaling pathway	13	3.01E-2
4068	FoxO signaling pathway	11	3.06E-2
4370	VEGF signaling pathway	7	3.62E-2
3040	Spliceosome	11	3.66E-2
4725	Cholinergic synapse	10	3.66E-2
4014	Ras signaling pathway	16	4.13E-2
4350	TGF-beta signaling pathway	8	4.13E-2
5016	Huntington s disease	14	4.13E-2
4015	Rap1 signaling pathway	15	4.78E-2
5142	Chagas disease (American trypanosomiasis)	9	4.79E-2
5211	Renal cell carcinoma	7	4.79E-2
3430	Mismatch repair	4	4.86E-2

Table S3: The details of top 15 significantly enriched KEGG pathways.

NO.	Pathway	Gene count	FDR	Matching proteins
1	Adherens junction	13	1.30E-4	CSNK2A2,CTNNA1,CTNNA3,EP300,FYN,LEF1,MAPK3,PVRL1,PVRL3,RHOA,SNAI2,TCF7L1,TJP1
2	Pathways in cancer	31	1.30E-4	AKT2,ARAF,BAX,BCL2,BCL2L1,BMP4,CDK4,CDK6,CKS2,CSF2RA,CSF3R,CTNNA1,CTNNA3,DVL3,EP300,FAS,FGF1,FGFR2,GLI3,HDAC1,LEF1,MAP2K1,MAPK3,MAX,PIK3CD,RB1,RHOA,STK4,TCF7L1,TFG,WNT7A
3	MicroRNAs in cancer	19	1.30E-4	BCL2,BCL2L2,CDK6,DNMT1,EP300,EZH2,EZR,HDAC1,HMGA2,MAP2K1,PLAU,RHOA,SHC1,SPRY2,STMN1,TPM3,TRIM71,UBE2I,VIM
4	MAPK signaling pathway	25	4.64E-3	AKT2,ARRB2,CACNA1G,CACNA2D1,CACNB1,FAS,FGF1,FGFR2,FLNA,MAP2K1,MAP3K1,MAP3K2,MAP3K4,MAPK3,MAPKAPK3,MAX,NR4A1,PAK2,PPP5C,RPS6KA1,RPS6KA3,RRAS,STK4,STMN1,TAOK3
5	Colorectal cancer	10	1.90E-3	AKT2,ARAF,BAX,BCL2,LEF1,MAP2K1,MAPK3,PIK3CD,RHOA,TCF7L1
6	Chronic myeloid leukemia	11	1.90E-3	AKT2,ARAF,BCL2L1,CDK4,CDK6,HDAC1,MAP2K1,MAPK3,PIK3CD,RB1,SHC1
7	HTLV-I infection	23	2.15E-3	AKT2,ATF3,BAX,BCL2L1,BUB3,CALR,CDC27,CDK4,DVL3,EP300,HLA-DRB1,MAP3K1,

				MYB,NRP1,PCNA,PIK3CD,POLD4,RB1,RRAS,SLC25A5,TBP,TBPL1,WNT7A
8	Endometrial cancer	9	2.15E-3	AKT2,ARAF,CTNNA1,CTNNA3,LEF1,MAP2K1,MAPK3,PIK3CD,TCF7L1
9	Glioma	10	2.15E-3	AKT2,ARAF,CAMK2G,CDK4,CDK6,MAP2K1,MAPK3,PIK3CD,RB1,SHC1
10	Prostate cancer	12	2.15E-3	AKT2,ARAF,BCL2,EP300,FGFR2,LEF1,MAP2K1,MAPK3,PDGFD,PIK3CD,RB1,TCF7L1
11	Cell cycle	14	2.94E-3	BUB3,CDC14A,CDC27,CDK4,CDK6,EP300,HDAC1,PCNA,PLK1,RB1,SMC1A,STAG2,TFDP1,YWHAQ
12	Oocyte meiosis	13	2.94E-3	CAMK2G,CDC27,MAP2K1,MAPK3,PLK1,PPP1CA,PPP2CB,PPP2R1A,PPP2R5B,RPS6KA1,RPS6KA3,SMC1A,YWHAQ
13	PI3K-Akt signaling pathway	27	2.94E-3	AKT2,BCL2,BCL2L1,CDK4,CDK6,COL5A1,COL6A3,CSF3R,CSH2,EIF4E2,EPHA2,FGF1,FGFR2,GNB2,ITGB8,KDR,MAP2K1,MAPK3,MYB,NR4A1,PDGFD,PIK3CD,PKN2,PPP2CB,PPP2R1A,PPP2R5B,YWHAQ
14	Non-small cell lung cancer	9	2.94 E-3	AKT2,ARAF,CDK4,CDK6,MAP2K1,MAPK3,PIK3CD,RB1,STK4
15	Melanoma	10	3.84E-3	AKT2,ARAF,CDK4,CDK6,FGF1,MAP2K1,MAPK3,PDGFD,PIK3CD,RB1

Note: FDR: False discovery rate

Supplementary Table S4: PubMed citations of the DE miRNA targets

mRNA	Associated with			Reference(PMID)
	chemoradiotherapy	rectal cancer	other cancers	
AKAP11	NO	NO	YES	19587378
ANKRD49	NO	NO	YES	19920108
ARIH2	NO	NO	YES	21454682
ARMC8	NO	NO	YES	28081738
ARPC4	NO	NO	YES	26648486
ARRB2	YES	NO	YES	22315403; 26273408
ATP1A1	YES	YES	YES	23910267;26835885;27845894
CAST	YES	YES	YES	15807633;15466976;27323818
CDC14A	YES	YES	YES	28386351;26747605;27323075
CYB5A	NO	NO	YES	25225034
DNER	NO	YES	YES	17143535; 19544453
EPHA2	YES	YES	YES	18339848;28165374;27804871
EPS8L3	NO	YES	YES	19223546; 19223546
FGFR2	YES	YES	YES	26831663;24968263;28430863
HIPK2	YES	YES	YES	21785465;25282590;28107201
HSD17B11	NO	NO	YES	21549806
IRF8	YES	YES	YES	27098631;25495942;26563595
IVNS1ABP	NO	NO	YES	25619834
KCNK1	NO	NO	YES	18239687
KCNMA1	NO	YES	YES	24367615; 28231797
KIAA0802	NO	NO	NO	NA
KLK10	NO	YES	YES	23499583; 27825132
MYO5A	NO	YES	YES	19521958; 21300763
NOL3	YES	YES	YES	21273594;21859567;26709830
PRG4	NO	NO	YES	28192118
PTPRD	NO	YES	YES	26847345; 28345455
RCE1	NO	NO	YES	26546252
RFC3	NO	YES	YES	20573375; 2788870
RGS12	YES	YES	YES	21698121;12461749;21209843
RUSC2	NO	NO	YES	27238570
SEMA6A	YES	YES	YES	15917651;17671748;17671748
SERF2	NO	NO	NO	NA
SF1	YES	YES	YES	28284009;17900258;17383426
SLC25A5	NO	NO	YES	19140237
SMUG1	YES	YES	YES	24253812;24036853;24229683
STAG2	YES	YES	YES	28169993;22668012;28302680
TBC1D17	NO	YES	YES	27217703; 24752605
TFAP2B	YES	YES	YES	20581741;22511254;24766673
TFDP1	YES	YES	YES	24598828;25133581;27871936

TIPARP	YES	NO	YES	26267320; 21712449
TMC7	YES	YES	NO	24316942; 24316942;
TMEM156	NO	NO	YES	26427334
TPST2	YES	NO	YES	11322664; 27992415
WTAP	YES	YES	YES	21150717;26071132;27370540
